# Bifunctional Catalysis: Direct Reductive Amination of Aliphatic Ketones with an Iridium-Phosphate Catalyst †

**DOI:** 10.3390/molecules15042453

**Published:** 2010-04-08

**Authors:** Barbara Villa-Marcos, Chaoqun Li, Keith R. Mulholland, Philip J. Hogan, Jianliang Xiao

**Affiliations:** 1 Liverpool Centre for Materials and Catalysis, Department of Chemistry, University of Liverpool, Liverpool L69 7ZD, UK; E-Mail: barvm@liv.ac.uk (B.V.M.); 2 AstraZeneca, Silk Road Business Park, Macclesfield, SK10 2NA, UK

**Keywords:** reductive amination, asymmetric hydrogenation, bifunctional catalyst, aliphatic ketones, chiral amines

## Abstract

Chiral amines are one of the ubiquitous functional groups in fine chemical, pharmaceutical and agrochemical products, and the most convenient, economical, and eco-benign synthetic pathway to these amines is direct asymmetric reductive amination (DARA) of prochiral ketones. This paper shows that a wide range of aliphatic ketones can be directly aminated under hydrogenation conditions, affording chiral amines with good to excellent yields and with enantioselectivities up to 96% ee. The catalysis is effected by the cooperative action of a cationic Cp*Ir(III) complex and its phosphate counteranion.

## 1. Introduction

**Figure 1:**
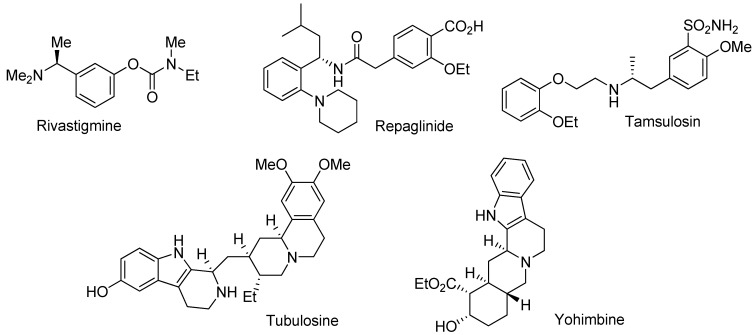
Examples of chiral amines in the pharmaceutical industry.

The use of enantiomerically pure compounds has steadily increased in the pharmaceutical and agrochemical industry in the past decades. About 80% of all drug candidates in the pipeline are now chiral [1]. Chiral amines in particular are important intermediates in the pharmaceutical chemistry. Representative examples include rivastigmine, a drug for the treatment of Alzeihmer’s [2], and repaglinide, which is used for the treatment of type II diabetes [3]. Tamsulosin, the blockbuster drug Flomax, can improve symptoms in patients with chronic prostatitis [4]. Additionally, chiral cyclic amines, such as the tetrahydro-β-carboline moiety frequently appear in natural products and biologically important molecules. For example, tubulosine displays high antitumor activity [5], and yohimbine is an antagonist for the serotonin 2B receptor [6] (Figure 1). 

Asymmetric imine hydrogenation [7,8,9,10,11,12,13,14,15,16] and direct asymmetric reductive amination [17,18,19] (DARA) provide convenient methods for the synthesis of chiral amines. The latter is especially attractive from a commercial point of view, since one step is saved, avoiding the tedious isolation of usually-unstable imine intermediates. However, there are only a few efficient homogeneous metal catalysts reported for DARA [20,21,22,23,24,25,26], and not surprisingly, DARA still remains a *key green chemistry research area* [27]. In particular, the DARA of aliphatic ketones is relatively unexplored when compared to that of aromatic ketones.

The first enantioselective reductive amination was reported by Blaser in 1999 for the synthesis of (*S*)-metolachlor, the active ingredient of an important grass herbicide [20]. The product is obtained in a reasonable 78% ee by the use of an Ir-xyliphos catalyst; but the scope of DARA has not been explored. Subsequently, Zhang and co-workers presented successful results for the DARA of aromatic ketones using an Ir-f-binaphane complex in the presence of Ti(O^i^Pr)_4_ and I_2_ [21]. However, this catalyst did not work for DARA of aliphatic ketones. Chiral amino acids have been prepared by DARA of the corresponding keto-esters/-acids [25,26]. Börner *et al*. reported the DARA of α-keto acids using a cationic Rh-Deguphos catalyst, affording good yields and up to 98% ee [25]. Subsequently, Bunlaksananusorn and Rampf reported the synthesis of β-amino esters with a Ru-ClMeOBIPHEP catalyst, obtaining 70-88% yields and excellent enantioselectivities (up to 99%) [26]. Recently, Rubio-Pérez *et al*. reported a successful catalyst for the DARA of aliphatic ketones [24]. The Pd-(*R*)-BINAP catalyst showed enantioselectivities up to 99%; however, it failed to give satisfactory results with aromatic ketones, where the ee was only up to 43%.

Transfer hydrogenation has also been used to effect DARA. Kadyrov and co-workers reported the synthesis of primary amines by DARA. Using the [Ru((*R*)-TolBINAP)Cl_2_] catalyst, excellent ee’s were achieved in the DARA of aromatic ketones. In contrast, only a 24% ee was observed in the case of an aliphatic ketone [22]. Wills and co-workers reported an intramolecular reductive amination under transfer hydrogenation conditions with a Ru(II)TsDPEN catalyst, affording a cyclic amine in 88% ee [23]. Taken together, these results show that there is a need for more versatile catalysts for DARA, which are active, enantioselective, and effective for both aromatic and aliphatic ketones.

**Scheme 1:**
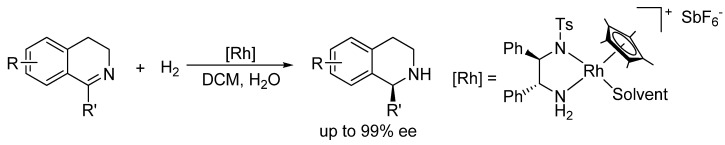
Hydrogenation of cyclic imines with a Rh(III)-chiral diamine catalyst.

Recently, we became interested in the asymmetric reduction of imines [28,29,30,31,32]. Under hydrogenation conditions, we obtained excellent results for the enantioselective reduction of cyclic imines with a cationic Rh(III)-diamine catalyst (Scheme 1) [29]. Unfortunately, when applied to acyclic imines the same catalyst was not effective, affording only poor ee’s.

**Figure 2:**
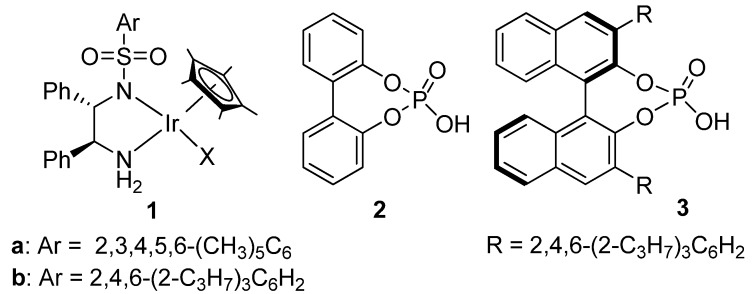
Catalyst **1** and phosphoric acids **2** and **3** used in this work.

Previously, the groups of Macmillan [33], Rueping [34] and List [35] have shown that chiral phosphoric acid catalysts such as **3** (Figure 2) can induce excellent ee´s in imine reduction and in DARA with Hantzsch esters, although the reaction times are generally long (up to 3 days). In these organocatalytic reactions, the phosphoric acid activates the imine via protonation and induces chirality by ion-pairing the resulting phosphate anion and iminium cation. Inspired by this work, we wondered whether a more versatile catalyst for DARA that uses H_2_ as the hydrogen could be constructed by the cooperative action of a H_2_-activating metal complex and a chiral phosphoric acid. 

**Scheme 2:**
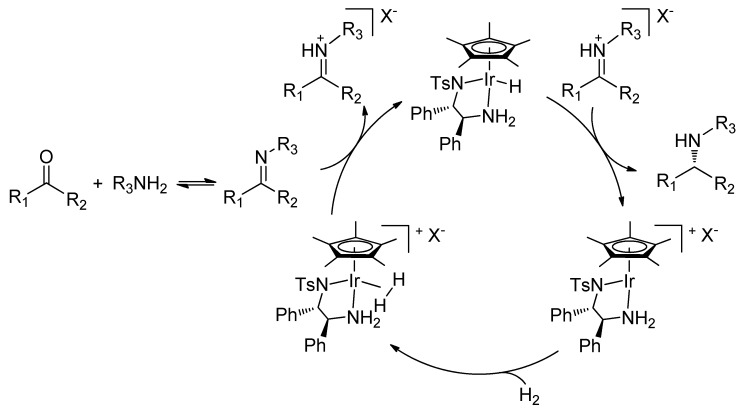
DARA of ketones via metal-counteranion cooperative catalysis.

The key elements of the cooperative catalysis are illustrated in Scheme 2. H_2_ is activated by the metal complex and heterolytically cleaved, forming an iminium cation by imine protonation and an Ir-hydride complex. In a non-polar solvent, the iminium cation ion-pairs, via hydrogen bonding, with the chiral phosphate anion [33,34,35], and is reduced by hydride transfer from the metal complex (Scheme 2). The protonation activates the C=N double bond towards attack by the hydride while ion pairing aids its enantiodiscrimination by the metal catalyst. This ionic hydrogenation pathway, which involves no coordination of the C=N double bond to the metal, has previously been demonstrated by Norton by using iminium tetrafluoroborate salts [36,37]. 

We first demonstrated the synergistic effect of the metal-counteranion combination in the asymmetric hydrogenation of acyclic imines [28]. Encouraged by these results, we turned our attention to DARA, successfully extending the concept of cooperative catalysis to the reductive amination of both aromatic and aliphatic ketones [30]. Herein, we describe the details of our work on the DARA of aliphatic ketones. 

## 2. Results and Discussion

### 2.1. Optimization of conditions

We started with optimization of the DARA conditions by considering the model reaction of 4-methyl-pentan-2-one (**4**) with *p*-anisidine. Table 1 summarizes the results. While the complex **1a** (X=Cl) (Figure 2) was inactive in the DARA of **4** (entry 1), exchange of the chloride for a non-coordinating counteranion such as SbF_6_^-^ and PF_6_^-^ allows the reaction to occur, but with low ee’s (15 and 35%, respectively; entries 2 and 3). Changing to the *pseudo*-chiral anion **2-H**, the enantioselectivity rose to 47% (entry 4). This prompted us to search for an Ir(III)-diamine complex containing a chiral counteranion. Thus, as reported before [28,30], when we moved to the chiral phosphate **3-H**, a 19% conversion and a much higher ee of 84% were observed in 6 h reaction time (entry 6). Aiming to boost the conversion and ee, we then studied the effect of extra acid **3** and molecular sieves on the DARA. As can be seen, additional **3** indeed resulted in a faster reaction; but its effect on the enantioselectivity was less significant (entries 6 *vs.* 7–9). In a similar way, the presence of molecular sieves (MS) increased the reaction rate but not the enantioselectivity (entries 6 *vs.* 10–13). We presume that the presence of the MS benefits the ketimine formation by removal of water from the reaction media. Furthermore, we were pleased to discover that in the presence of the MS, the acid **3** is no longer critical to the DARA rate and ee (entry 12 *vs.* 16), an observation that is in contrast to what was observed in the DARA of aromatic ketones [30]. 

**Table 1:** Optimisation of conditions for the DARA of 4-methylpentan-2-one (**4**).^a^ 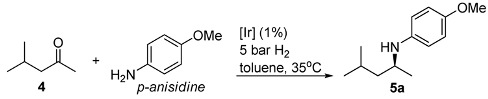

Entry	Ar	X	Addtive	Conv. (%) ^b^	Ee(%) ^c^
1	**a**	Cl	-	N.R. (17 h)	-
2	**a**	Cl	AgSbF_6_ (2%)	42 (17 h)	15 *(R)*
3	**a**	Cl	AgPF_6 _(2%)	60 (17 h)	35 *(R)*
4	**a**	**2-H**	-	11 (17 h)	47 *(R)*
5	**a**	**2-H**	**2** (5%)	28 (17 h)	46 *(R)*
6	**a**	**3-H**	-	19	84 *(S)*
7	**a**	**3-H**	**3** (1%)	42	85 *(S)*
8	**a**	**3-H**	**3** (5%)	60	86 *(S)*
9	**a**	**3-H**	**3** (8%)	67	89 *(S)*
10	**a**	**3-H**	4Å MS (50 mg)	30	85 *(S)*
11	**a**	**3-H**	4Å MS (100 mg)	50	86 *(S)*
12	**a**	**3-H**	4Å MS	59	86 *(S)*
13	**a**	**3-H**	4Å MS (200 mg)	59	86 *(S)*
14	**a**	**3-H**	**3** (1%),4Å MS	58	85 *(S)*
15 ^d^	**a**	**3-H**	**3** (5%),4Å MS	63	86 *(S)*
16	**a**	**3-H**	**3** (8%),4Å MS	72	86 *(S)*
17	**a**	**3-H**	4Å MS	57 (24 h)	91 *(S)*
18	**b**	**3-H**	-	5	-
19	**b**	**3-H**	4Å MS	7	-
20	**b**	**3-H**	**3** (8%)	50	71 *(S)*
21	**b**	**3-H**	**3** (8%),4Å MS	60	74 *(S)*
22	**a**	**3-H**	4Å MS	>99 (12 h)	87 *(S)*

*^a^ Reaction conditions*: 0.55 mmol of **4**, 0.5 mmol of *p*-anisidine, 1 mol% of catalyst, 2 mL of toluene, 5 bar of H_2_, 35 ºC, 150 mg of 4 Å MS when added unless specified, 6 h unless specified. ^b^ Conversion of *p*-anisidine, determined by ^1^H-NMR analysis of the crude product. ^c^ Determined by HPLC analysis; ^d ^20 ºC.

**Scheme 3:**
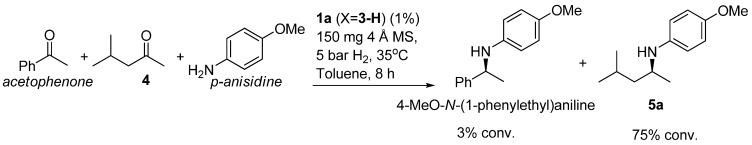
Competitive DARA of an aromatic and an aliphatic ketone with *p*-anisidine.

The observations above point to an easier ketimine formation in the DARA of aliphatic ketones. In the case of aromatic ketones, the reaction appears to be limited by this step and hence necessitates both a Brønsted acid and MS, which can catalyze the ketone-amine condensation and help shift the resulting equilibrium by removing water [28,30]. Scheme 3 illustrates a competition reaction between an aromatic and an aliphatic ketone, which led to the predominant DARA of **4**, showing that aromatic ketones are essentially inactive under the questions employed. When dealing with the aliphatic ketones, we also noted that there was no competition between hydrogenation of ketones over imines; the ketones were not reduced under the DARA conditions.

A quick screening on the ligand showed that catalyst **1a** (X = **3-H**) surpassed **1b** (X = **3-H**) in terms of activity and enantioselectivity (entry 16 *vs.* 21). In addition, the effect of temperature was also studied. Although a slight increase in the enantioselectivity was observed (entry 17), lowering the temperature to 20 °C afforded only a 57% conversion after 24 h. Finally, we confirmed the reaction goes to completion in 12 h reaction time (entry 22). Taken together, these results suggest the optimum reaction conditions to include catalyst **1a** (X = **3-H**) and MS (150 mg), and we deemed the addition of extra **3** unnecessary, avoiding the use of an expensive chiral acid.

### 2.2. Scope of substrates

Next, we explored the application of the optimized reaction conditions to a variety of aliphatic ketones and aromatic amines. As can be seen, a wide range of aliphatic ketones were readily aminated with *p*-anisidine (Table 2), *m*-anisidine (Table 3) and relatively more electron-deficient anilines (Table 4) under 5 bar of H_2 _at 35 ºC. The amines **5, 6 **and **7** were obtained with good yields and excellent enantioselectivities in general. Notably, the catalyst tolerates other reducible functional groups in the substrates, such as terminal (**5i**) and internal alkenes (**5j**, **6h**, **7e**) and highly strained cyclopropyl ring (**5h**, **6i**, **7f**). 

Table 2 presents the results for the DARA with *p*-anisidine. Compared to our results reported for imine hydrogenation at 20 ºC (**5a**, **5e**, **5i**) [28], the enantioselectivity is slightly lower, but with the advantage of not isolating the imines. The product **5c** was previously obtained in 84% ee [38], via the reduction of the corresponding enantiomerically pure α-sulfinyl ketimine followed by desulfinylation. **5e** appeared in the DARA with the Pd-(*R*)-BINAP, showing a slightly lower ee (76%) [24]. The amine **5i** was obtained by Macmillan et al. in 90% ee in DARA with Hanztsch ester, but requiring a long reaction time (96 h) [33]. Finally, the amine **5j** has also been reported, as the product of the hydrosilylation of the corresponding imine with 90% ee [39]. 

In general, the products arising from the DARA of alkyl-methyl ketones with *p*-anisidine are obtained with good yields and excellent ee’s. However, the enantioselectivity dropped significantly when alkyl-ethyl ketones were aminated (**5f**, **5g**). Clearly, the catalyst is unable to discriminate efficiently an ethyl from a butyl or pentyl group.

**Table 2:** DARA of aliphatic ketones with *p*-anisidine.^a^

Entry	Product	Yield (%)	Ee ^b^ (%)	Entry	Product	Yield (%)	Ee ^b^ (%)
1	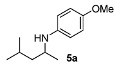	91	87	7	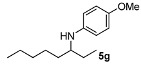	80	71
2	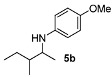	82	96 ^c^	8	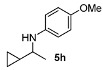	90	93
3	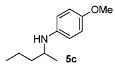	88	90	9	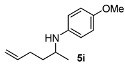	80	92
4	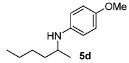	82	93	10	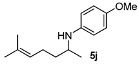	89	95
5	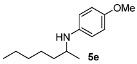	79	91	11	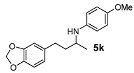	85	93
6	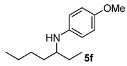	80	49				

*^a ^Reaction conditions*: 0.55 mmol of ketone, 0.5 mmol of *p*-anisidine, 1 mol% of **1a (**X =**3-H)**, 2 mL of toluene, 150 mg of 4 Å MS, 5 bar of H_2_, 35 °C, 12–20 h. ^b^ Determined by HPLC; *S* configuration, assigned by comparison with the literature [33]. ^c^ ~2% d.e.

With the sterically more demanding *m*-methoxyanilines, both the yields and ee’s remained good, although lower than those with *p*-anisidines in most cases (Table 3). This lowering in ee may stem from a less favored interaction of the iminium ion with either the metal catalyst or the counteranion or both. Worthy of particular mention is the amine **6c**, where the catalyst is capable of differentiating an isopropyl and a methyl group, affording an excellent 90% ee. The amines **6e**, **6h** and **6i** have been previously reported as products for a tandem intermolecular hydroamination-transfer hydrogenation of alkynes [40]. Although similar enantioselectivities were achieved, much longer reaction times appear to be necessary (up to 72 h). As in the case of *p*-anisidines, the enantioselectivity dropped significantly when alkyl-ethyl ketones were used (**6f **and **6g**).

**Table 3:** DARA with *m*-substituted anilines.^a^

Entry	Product	Yield (%)	Ee (%)	Entry	Product	Yield (%)	Ee (%)
1	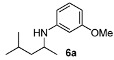	72	80	6	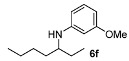	81	61
2	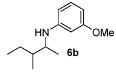	82	96 ^b^	7	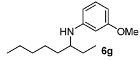	83	64
3	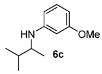	86	90	8	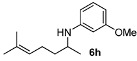	67	82
4	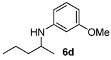	77	91	9	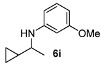	62	82
5	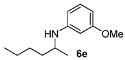	85	92	10	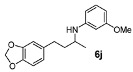	80	91

^a^ Reaction conditions were the same as those in Table 2, except with 0.5 mmol *m*-anisidine. ^b^ ~8% d.e.

We then investigated DARA of aliphatic ketones with aniline and more electron-deficient analogues. Amines **7** were obtained with good yields and enantioselectivities (Table 4). Of particular note is that 4-bromo aniline also reacted, giving rise to a decent yield and ee, albeit in a longer reaction time (**7h**). Other aliphatic ketones were also aminated with 4-bromo as well as 4-chloro-aniline, affording 70–90% yields. However, we have not been able to find suitable conditions to separate the enantiomers by HPLC; so they are not included in this paper. Compounds **7a** and **7b** were previously obtained in DARA with the [PdBr_2_((*R*)-BINAP)] catalyst [24], with only a 51% and 10% ee, respectively. A poor ee (17%) was also observed for the amine **7b** in the hydrogenation of the corresponding imine with a cationic Ir(I) complex containing a chiral phosphanodihydrooxazole ligand [41]. Similarly, the amine **7d** was previously obtained in only 18% ee in the literature [42]. The current catalytic system does not work with aliphatic amines, however, where the intermediate imine is formed but not reduced.

**Table 4:** DARA with aniline and 4-bromoaniline.^a^

Entry	Product	Yield (%)	Ee (%)	Entry	Product	Yield (%)	Ee (%)
1	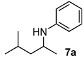	80	88	5	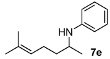	91	91
2	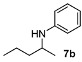	92	94	6	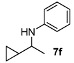	91	92
3	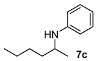	83	95	7	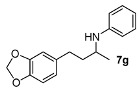	95	91
4	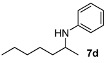	83	92	8 ^b^	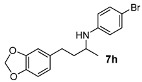	70	84

*^a^* Same conditions as those in Table 2, except with 0.5 mmol aniline. ^b^ 0.5 mmol 4-bromoaniline, 30 h.

**Figure 3:**
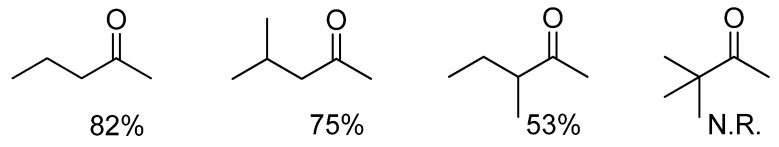
Conversions observed in the DARA with **1a** (X = **3-H**) (1%) at 5 bar H_2_ and 35 ºC in 8 h.

To further appreciate how steric effects affect the cooperative DARA, we compared the amination of the ketones in Figure 3 with *p*-anisidine. As can be seen, with increasing steric hindrance near the carbonyl carbon, the DARA becomes slower, and no amination was observed with the sterically most demanding *tert*-butylmethyl ketone, even after 24 h. This reduction in rate is most likely a result of increased difficulty in hydride transfer. The enantioselectivity is, however, more subtle to explain, as it does not follow the trend of reaction rate and is highest with a sterically bulky ketone (entries 2 *vs*. 1 and 3, Table 2). This is probably a reflection of the enantioselectivity being controlled by both the Ir(III) catalyst and its counteranion. The former is responsible for hydride delivery and would be expected to respond to steric effects in the manner observed. 

## 3. Experimental

### 3.1. General

Unless otherwise specified, the chemicals were obtained commercially and used without further purification. Toluene was dried over sodium and distilled prior to use. 4 Å molecular sieves (MS) were dried in an oven at 160 ºC for a minimum of 24 h. NMR spectra were recorded on a DRX-400 spectrometer operating at 400 MHz (^1^H) or 100 Mhz (^13^C) using CDCl_3_ as solvent and TMS as the internal standard. The mass spectra were obtained by chemical ionization (CI). HPLC analysis was performed on a Gilson UV/VIS-151 equipped with an OD-H, OB-H or OJ column purchased from Daicel Chemical Industries. The catalysts **1a **(X = **2-H**), **1a **(X = **3-H**) and **1b **(X = **3-H**) were synthesized according to the literature procedure by reacting the corresponding 16 e^- ^species with the phosphoric acid **2 **or **3**, without further purification [28,43]. Compounds **2** and **3** were also prepared following the literature procedure [33,35], as well as catalyst **1a** (X = Cl) [44]. The configuration of **5i** was assigned by comparison with the literature [33], and that of the rest was based on analogy with the assignment for that compound without verification.

### 3.2. General procedure for DARA

To a glass liner equipped with a stir bar was added 4 Å MS (150 mg), aliphatic ketone (0.55 mmol), amine (0.5 mmol), catalyst (5 μmol) and distilled toluene (2 mL). The glass liner was then placed into an autoclave, followed by degassing with H_2_ three times. The hydrogenation was carried out at 5 bar H_2_ with stirring at 35 ºC for 12–30 h. The hydrogen gas was then carefully released in a fume hood and the solution was filtered, transferred to a flask, and concentrated to afford the crude product. Flash chromatography purification with a column of silica gel eluted with petroleum ether/ethyl acetate (15/1) yielded the desired amine product.

*4-Methoxy-N-(4-methylpentan-2-yl)aniline* (**5a**) [28]. The product (94 mg, 91% yield, 87% ee) was obtained according to the general procedure from 4-methylpentan-2-one (55 mg, 0.55 mmol) and *p*-anisidine (62 mg, 0.5 mmol) in 12 h; ^1^H-NMR: δ 0.83 (d, *J* = 6.6 Hz, 3H), 0.85 (d, *J*= 6.6 Hz, 3H), 1.05 (d, *J* = 6.3 Hz, 3H), 1.15 (dt, *J* = 13.7, 6.9 Hz, 1H), 1.37 (dt, *J* = 13.7, 6.9 Hz, 1H), 1.62–1.72 (m, 1H), 2.92 (brs, 1H), 3.31-3.39 (m, 1H), 3.66 (s, 3H), 6.45–6.49 (m, 2H), 6.67–6.71 (m, 2H); ^13^C-NMR: δ 21.5, 22.9, 23.4, 25.5, 47.4, 48.0, 56.2, 115.1, 115.4, 142.3, 152.2; HRMS for C_13_H_22_NO [M+H]^+^: *m/z* Calcd: 208.1701; Found: 208.1705; HPLC (Chiralcel OD-H, hexane:isopropanol = 99:1, flow rate 0.5 mL/min, λ = 254 nm): t_R_ = 13.5 min (minor), t_R_ = 14.6 min (major). 

*4-Methoxy-N-(3-methylpentan-2-yl)aniline* (**5b**). Mixture of diastereoisomers 51:49. The product (85 mg, 82% yield, 96% ee) was obtained according the general procedure from 3-methylpentan-2-one (55 mg, 0.55 mmol) and *p*-anisidine (62 mg, 0.5 mmol) in 20 h; ^1^H-NMR: δ 0.84–0.94 (m, 6H), 1.03 (d, *J* = 8.6 Hz, 1.5H), 1.07 (d, *J* = 7.6 Hz, 1.5H), 1.13–1.25 (m, 1H), 1.42–1.63 (m, 2H), 3.31–3.40 (m, 1H), 3.74 (s, 3H), 6.55–6.58 (m, 2H), 6.74–6.78 (m, 2H); ^13^C-NMR: δ 12.3(7), 12.4(3), 14.0, 15.7, 16.0, 17.6, 25.2, 27.0, 38.6, 39.5, 53.5, 54.1, 56.2, 115.2, 115.4, 142.5, 152.2; HRMS for C_13_H_22_NO [M+H]^+^: *m/z* Calcd: 208.1701; Found: 208.1697; HPLC (Chiralcel OD-H, hexane:isopropanol = 99:1, flow rate 0.5 mL/min, λ = 254 nm): t_R_ = 12.0 min (d1, minor), t_R_ = 12.9 min (d2, minor), t_R_ = 13.8 (d1, major), t_R_ = 14.8 (d2, major).

*4-Methoxy-N-(pentan-2-yl)aniline* (**5c**) [38]. The product (85 mg, 88% yield, 90% ee) was obtained according to the general procedure from 2-pentanone (47 mg, 0.55 mmol) and *p*-anisidine (62 mg, 0.5 mmol) in 20 h; ^1^H-NMR: δ 0.92 (t, *J* = 7.1 Hz, 3H), 1.14 (d, *J* = 6.3 Hz, 3H), 1.33–1.45 (m, 3H), 1.50–1.57 (m, 1H), 3.11 (brs, 1H), 3.38 (sextet, *J* = 6.3 Hz, 1H), 3.74 (s, 3H), 6.53–6.57 (m, 2H), 6.75–6.79 (m, 2H); ^13^C-NMR δ 14.6, 19.8, 21.2, 39.9, 49.6, 56.2, 115.1, 115.4, 142.4, 152.2; HRMS for C_12_H_20_NO [M+H]^+^: *m/z* Calcd: 194.1545; Found: 194.1539; HPLC (Chiralcel OD-H, hexane:isopropanol = 99:1, flow rate 0.5 mL/min, λ = 254 nm): t_R_ = 15.1 min (minor), t_R_ = 15.8 min (major). 

*4-Methoxy-N-(hexan-2-yl)aniline* (**5d**) [45]. The product (85 mg, 82% yield, 93% ee) was obtained according to the general procedure from 2-hexanone (55 mg, 0.55 mmol) and *p*-anisidine (62 mg, 0.5 mmol) in 20 h; ^1^H-NMR: δ 0.90 (t, *J* = 7.1 Hz, 3H), 1.15 (d, *J* = 6.3 Hz, 3H), 1.30–1.42 (m, 5H), 1.53–1.59 (m, 1H), 3.36 (sextet, *J* = 6.3 Hz, 1H), 3.74 (s, 3H), 6.53–6.57 (m, 2H), 6.75–6.79 (m, 2H); ^13^C-NMR: δ 14.5, 21.2, 23.2, 28.8, 37.4, 49.9, 56.3, 115.1, 115.4, 142.5, 152.2; HRMS for C_13_H_22_NO [M+H]^+^: *m/z* Calcd: 208.1701; Found: 208.1705; HPLC (Chiralcel OD-H, hexane:isopropanol = 99:1, flow rate 0.5 mL/min, λ = 254 nm): t_R_ = 14.1 min (minor), t_R_ = 14.7 min (major).

*N-(Heptan-2-yl)-4-methoxyaniline* (**5e**) [24,28]. The product (87 mg, 79% yield, 91% ee) was obtained according to the general procedure from 2-heptanone (63 mg, 0.55 mmol) and *p*-anisidine (62 mg, 0.5 mmol) in 20 h; ^1^H-NMR: δ 0.89 (t, *J* = 6.8 Hz, 3H), 1.14 (d, *J* = 6.3 Hz, 3H), 1.24–1.32 (m, 4H), 1.34–1.43 (m, 3H), 1.50–1.59 (m, 1H), 3.12 (brs, 1H), 3.32–3.39 (m, 1H), 3.74 (s, 3H), 6.53–6.57 (m, 2H), 6.75–6.79 (m, 2H); ^13^C-NMR: δ 14.4, 21.2, 23.1, 26.3, 32.3, 37.6, 50.0, 56.3, 115.1, 115.4, 142.4, 152.3; HRMS for C_14_H_24_NO [M+H]^+^:*m/z* Calcd: 222.1858; Found: 222.1852; HPLC (Chiralcel OB-H, hexane:isopropanol = 99:1, flow rate 1.0 mL/min, λ = 254 nm): t_R_ = 9.3 min (major), t_R_ = 10.6 min (minor).

*N-(Heptan-3-yl)-4-methoxyaniline* (**5f**). The product (105 mg, 95% yield, 49% ee) was obtained according to the general procedure from 3-heptanone (63 mg, 0.55 mmol) and *p*-anisidine (62 mg, 0.5 mmol) in 20 h; ^1^H-NMR: δ 0.89 (t, *J* = 7.1 Hz, 3H), 0.91 (t, *J* = 7.5 Hz, 3H), 1.26–1.59 (m, 8H), 3.18 (quintet, *J* = 6.0 Hz, 1H), 3.74 (s, 3H), 6.52-6.56 (m, 2H), 6.74–6.78 (m, 2H); ^13^C-NMR δ 10.4, 14.5, 23.3, 27.5, 28.6, 34.4, 55.5, 56.3, 114.7, 115.4, 142.9, 151.9; HRMS for C_14_H_24_NO [M+H]^+^: *m/z* Calcd: 222.1858; Found: 222.1850; HPLC (Chiralcel OB-H, hexane:isopropanol = 99:1, flow rate 0.5 mL/min, λ = 254 nm): t_R_ = 14.0 min (major), t_R_ = 15.8 min (minor).

*4-Methoxy-N-(octan-3-yl)aniline* (**5g**). The product (94 mg, 80% yield, 71% ee) was obtained according to the general procedure from 3-octanone (71 mg, 0.55 mmol) and *p*-anisidine (62 mg, 0.5 mmol) in 20 h; ^1^H-NMR: δ 0.86–0.93 (m, 6H), 1.27–1.59 (m, 10H), 3.14 (brs, 1H), 3.18 (quintet, *J* = 5.9 Hz, 1H), 3.74 (s, 3H), 6.54 (d, *J* = 8.8 Hz, 2H), 6.76 (d, *J* = 8.8 Hz, 2H); ^13^C-NMR: δ 10.4, 14.5, 23.1, 26.1, 27.5, 32.5, 34.7, 55.5, 56.3, 114.7, 115.3, 142.9, 151.9; HRMS for C_15_H_26_NO [M+H]^+^: *m/z* Calcd: 236.2009; Found: 236.2006; HPLC (Chiralcel OB-H, hexane:isopropanol = 99:1, flow rate 1.0 mL/min, λ = 254 nm): t_R_ = 11.0 min (major), t_R_ = 13.6 min (minor).

*N-(1-Cyclopropylethyl)-4-methoxyaniline* (**5h**). The product (86 mg, 90% yield, 93% ee) was obtained according to the general procedure from 1-cyclopropylethanone (46 mg, 0.55 mmol) and *p*-anisidine (62 mg, 0.5 mmol) in 20 h; ^1^H-NMR: δ 0.21–0.31 (m, 2H), 0.43–0.52 (m, 2H), 0.86–0.95 (m, 1H), 1.20 (d, *J* = 6.3 Hz, 3H), 2.82-2.89 (m, 1H), 3.74 (s, 3H), 6.56–6.60 (m, 2H), 6.74–6.78 (m, 2H); ^13^C- NMR: δ 2.9, 3.6, 18.4, 20.7, 54.2, 56.2, 115.2, 115.5, 142.6, 152.3; HRMS for C_12_H_18_NO [M+H]^+^: *m/z* Calcd: 192.1388; Found: 192.1381; HPLC (Chiralcel OB-H, hexane:isopropanol = 99:1, flow rate 0.5 mL/min, λ = 254 nm): t_R_ = 18.4 min (major), t_R_ = 24.4 min (minor). 

*N-(Hex-5-en-2-yl)-4-methoxyaniline* (**5i**) [28,33]. The product (82 mg, 80% yield, 92% ee) was obtained according to the general procedure from hex-5-en-2-one (54 mg, 0.55 mmol) and *p*-anisidine (62 mg, 0.5 mmol) in 20 h; ^1^H-NMR: δ 1.15 (d, *J* = 6.3 Hz, 3H), 1.38–1.47 (m, 1H), 1.54–1.63 (m, 1H), 2.05–2.11 (m, 2H), 3.10 (brs, 1H), 3.33 (sextet, *J* = 6.3 Hz, 1H), 3.67 (s, 3H), 4.88–4.92 (m, 1H), 4.96 (dq, *J* = 17.0, 1.7 Hz, 1H), 5.76 (ddt, *J* = 17.0, 10.3, 6.7 Hz, 1H), 6.46–6.50 (m, 2H), 6.68–6.72 (m, 2H); ^13^C-NMR: δ 21.2, 30.9, 36.7, 49.4, 56.2, 115.1, 115.2, 115.4, 138.9, 142.3, 152.3; HRMS for C_13_H_20_NO [M+H]^+^: *m/z* Calcd: 206.1545; Found: 206.1547; HPLC (Chiralcel OB-H, hexane:isopropanol = 99:1, flow rate 1.0 mL/min, λ = 254 nm): t_R_ = 13.4 min (major), t_R_ = 15.8 min (minor).

*4-Methoxy-N-(6-methylhept-5-en-2-yl)aniline* (**5j**) [39]. The product (104 mg, 89% yield, 95% ee) was obtained according to the general procedure from 6-methylhept-5-en-2-one (69 mg, 0.55 mmol) and *p*-anisidine (62 mg, 0.5 mmol) in 20 h; ^1^H-NMR: δ 1.16 (d, *J* = 6.3 Hz, 3H), 1.39–1.48 (m, 1H), 1.59 (s, 3H), 1.54–1.63 (m, 1H), 1.69 (d, *J* = 0.8 Hz, 3H), 2.05–2.10 (m, 2H), 3.37 (sextet, *J* = 6.3 Hz, 1H), 3.74 (s, 3H), 5.10–5.14 (m, 1H), 6.53–6.57 (m, 2H), 6.75–6.79 (m, 2H); ^13^C-NMR: δ 18.1, 21.2, 25.1, 26.1, 37.6, 49.5, 56.2, 115.1, 115.3, 124.5, 132.4, 142.5, 152.3; HRMS for C_15_H_24_NO [M+H]^+^: *m/z* Calcd: 234.1858; Found: 234.1865; HPLC (Chiralcel OB-H, hexane:isopropanol = 99:1, flow rate 0.5 mL/min, λ = 254 nm): t_R_ = 21.6 min (major), t_R_ = 26.9 min (minor). 

*N-[4-(2H-1,3-benzodioxol-5-yl)butan-2-yl]-4-methoxyaniline* (**5k**). The product (127 mg, 85% yield, 93% ee) was obtained according to the general procedure from 4-benzo[1,3]dioxol-5-yl-butan-2-one (106 mg, 0.55 mmol) and *p*-anisidine (62 mg, 0.5 mmol) in 20 h; ^1^H-NMR: δ 1.18 (d, *J* = 6.4 Hz, 3H), 1.65–1.74 (m, 1H), 1.78–1.87 (m, 1H), 2.64 (t, *J* = 7.9 Hz, 2H), 3.36–3.41 (m, 1H), 3.75 (s, 3H), 5.92 (s, 2H), 6.53 (d, *J* = 8.5 Hz, 2H), 6.62 (d, *J* = 7.8 Hz, 1H), 6.67 (d, *J* = 1.6 Hz, 1H), 6.72 (d, *J* = 7.8 Hz, 1H), 6.75–6.77 (m, 2H); ^13^C-NMR: δ 21.3, 32.6, 39.5, 49.3, 56.2, 101.2, 108.6, 109.3, 115.2, 115.4, 121.6, 136.4, 142.1, 146.0, 148.0, 152.3; HRMS for C_18_H_22_NO_3_ [M+H]^+^: *m/z* Calcd: 300.1599; Found: 300.1590; HPLC (Chiralcel OD-H, hexane:isopropanol = 98:2, flow rate 1 mL/min, λ = 254 nm): t_R_ = 28.7 min (major), t_R_ = 38.6 min (minor).

*3-Methoxy-N-(4-methylpentan-2-yl)aniline* (**6a**). The product (75 mg, 72% yield, 80% ee) was obtained according the general procedure from 4-methyl-2-pentanone (55 mg, 0.55 mmol) and *m*-anisidine (62 mg, 0.5 mmol) in 20 h; ^1^H-NMR: δ 0.90 (d, *J* = 6.6 Hz, 3H), 0.94 (d, *J* = 6.6 Hz, 3H), 1.15 (d, *J* = 6.8 Hz, 3H), 1.25 (dt, *J* = 13.6, 6.8 Hz, 1H), 1.46 (dt, *J* = 13.6, 6.9 Hz, 1H), 1.71–1.79 (m, 1H), 3.40 (brs, 1H), 3.46–3.54 (m, 1H), 3.76 (s, 3H), 6.13 (t, *J* = 2.2 Hz, 1H), 6.21 (dd, *J* = 8.1, 2.2 Hz, 2H), 7.06 (t, *J* = 8.1 Hz, 1H); ^13^C-NMR: δ 21.5, 23.0, 23.4, 25.5, 47.0, 47.3, 55.5, 99.3, 102.2, 106.7, 130.4, 149.5, 161.3; HRMS for C_13_H_22_NO [M+H]^+^: *m/z* Calcd: 208.1701; Found: 208.1711; HPLC (Chiralcel OD-H, hexane:isopropanol = 98:2, flow rate 0.5 mL/min, λ = 254 nm): t_R_ = 19.9 min (minor), t_R_ = 21.9 min (major). 

*3-Methoxy-N-(3-methylpentan-2-yl)aniline* (**6b**). Mixture of diastereoisomers 54:46. The product (85 mg, 82% yield, 96% ee) was obtained according the general procedure from 3-methylpentan-2-one (55 mg, 0.55 mmol) and *m*-anisidine (62 mg, 0.5 mmol) in 20 h; ^1^H-NMR: δ 0.86 (d, *J* = 6.8 Hz, 1.5H), 0.89 (t, *J* = 7.4 Hz, 1.5H), 0.82–0.96 (m, 3H), 1.06 (d, *J* = 6.5 Hz, 1.5H), 1.10 (d, *J* = 6.5 Hz, 1.5H), 1.13–1.23 (m, 1H), 1.44–1.65 (m, 2H), 3.40–3.45 (m, 1H), 3.53 (brs, 1H), 3.76 (s, 3H), 6.13 (t, *J* = 2.2 Hz, 1H), 6.18-6.23 (m, 2H), 7.03–7.07 (m, 1H); ^13^C-NMR: δ 12.3(6), 12.4(3), 14.2, 15.7, 16.3, 17.7, 25.3, 26.9, 38.9, 39.6, 52.1, 52.7, 55.5, 99.2, 99.3, 101.9, 102.0, 106.6,(3), 106.6(7), 130.4, 149.5, 149.7, 161.3; HRMS for C_13_H_22_NO [M+H]^+^: *m/z* Calcd: 208.1701; Found: 208.1700; HPLC (Chiralcel OD-H, hexane:isopropanol = 99.5:0.5, flow rate 0.5 mL/min, λ = 254 nm): t_R_ = 46.7 min (d1, major), t_R_ = 50.3 min (d2, major), t_R_ = 55.6 (d1, minor), t_R_ = 58.2 (d2, minor).

*3-Methoxy-N-(3-methylbutan-2-yl)aniline* (**6c**). The product (83 mg, 86% yield, 90% ee) was obtained according the general procedure from 3-methylbutan-2-one (47 mg, 0.55 mmol) and *m*-anisidine (62 mg, 0.5 mmol) in 20 h; ^1^H-NMR: δ 0.90 (d, * J* = 6.8 Hz, 3H), 0.96 (d, *J* = 8.0 Hz, 3H), 1.09 (d, *J* = 6.5 Hz, 3H), 1.80–1.88 (m, 1H), 3.28–3.42 (m, 1H), 3.52 (brs, 1H), 3.76 (s, 3H), 6.13 (t, *J* = 2.3 Hz, 1H), 6.18–6.23 (m, 2H), 7.05 (t, *J* = 8.1 Hz, 1H); ^13^C-NMR: δ 17.0, 17.9, 19.6, 32.6, 53.8, 55.5, 99.3, 102.0, 106.7, 130.4, 149.6, 161.3; HRMS for C_12_H_20_NO [M+H]^+^: *m/z* Calcd: 194.1539; Found: 194.1541; HPLC (Chiralcel OB-H, hexane:isopropanol = 98:2, flow rate 0.5 mL/min, λ = 254 nm): t_R_ = 10.9 min (minor), t_R_ = 12.9 min (major).

*3-Methoxy-N-(pentan-2-yl)aniline* (**6d**). The product (74 mg, 77% yield, 91% ee) was obtained according the general procedure from 2-pentanone (47 mg, 0.55 mmol) and *m*-anisidine (62 mg, 0.5 mmol) in 20 h; ^1^H-NMR: δ 0.92 (t, *J* = 7.1 Hz, 3H), 1.16 (d, *J* = 6.3 Hz, 3H), 1.36–1.43 (m, 3H), 1.52–1.57 (m, 1H), 3.41–3.48 (m, 2H), 3.77 (s, 3H), 6.13 (t, *J* = 2.3 Hz, 1H), 6.19 (ddd, *J* = 8.1, 2.3, 0.8 Hz, 1H), 6.23 (ddd, *J* = 8.1, 2.3, 0.8 Hz, 1H), 7.06 (t, *J* = 8.1 Hz, 1H); ^13^C-NMR: δ 14.5, 19.7, 21.2, 39.8, 48.7, 55.5, 99.5, 102.3, 106.8, 130.4, 149.4, 161.3; HRMS for C_13_H_22_NO [M+H]^+^: *m/z* Calcd: 194.1539; Found: 194.1540; HPLC (Chiralcel OB-H, hexane:isopropanol = 98:2, flow rate 0.5 mL/min, λ = 254 nm): t_R_ = 14.4 min (minor), t_R_ = 16.0 min (major).

*N-(Hexan-2-yl)-3-methoxyaniline* (**6e**) [40]. The product (88 mg, 85% yield, 92% ee) was obtained according the general procedure from 2-hexanone (55 mg, 0.55 mmol) and *m*-anisidine (62 mg, 0.5 mmol) in 20 h; ^1^H-NMR: δ 0.90 (t, *J* = 7.1 Hz, 3H), 1.16 (d, *J* = 6.3 Hz, 3H), 1.30–1.45 (m, 5H), 1.52-1.58 (m, 1H), 3.43 (sextet, *J* = 6.3 Hz, 1H), 3.46 (brs, 1H), 3.77 (s, 3H), 6.13 (t, *J* = 2.3 Hz, 1H), 6.18-6.24 (m, 2H), 7.06 (t, *J* = 8.1 Hz, 1H); ^13^C-NMR: δ 14.5, 21.2, 23.2, 28.8, 37.3, 49.0, 55.5, 99.5, 102.3, 106.8, 130.4, 149.3, 161.3; HRMS for C_13_H_22_NO [M+H]^+^: *m/z* Calcd: 208.1696; Found: 208.1696; HPLC (Chiralcel OB-H, hexane:isopropanol = 98:2, flow rate 0.5 mL/min, λ = 254 nm): t_R_= 13.5 min (minor), t_R_ = 15.5 min (major).

*N-(Heptan-3-yl)-3-methoxyaniline* (**6f**). The product (90 mg, 81% yield, 61% ee) was obtained according the general procedure from 3-heptanone (63 mg, 0.55 mmol) and *m*-anisidine (62 mg, 0.5 mmol) in 20 h; ^1^H-NMR: δ 0.89 (t, *J* = 7.0 Hz, 3H), 0.91 (t, *J* = 7.4 Hz, 3H), 1.24–1.63 (m, 8H), 3.25 (quintet, *J* = 6.0 Hz, 1H), 3.45 (brs, 1H), 3.77 (s, 3H), 6.12 (t, *J* = 2.2 Hz, 1H), 6.17–6.22 (m, 2H), 7.05 (t, *J* = 8.1 Hz, 1H); ^13^C-NMR: δ 10.5, 14.5, 23.3, 27.7, 28.6, 34.5, 54.5, 55.5, 99.1, 101.8, 106.5, 130.4, 150.0, 161.3; HRMS for C_14_H_24_NO [M+H]^+^: *m/z* Calcd: 222.1852; Found: 222.1851; HPLC (Chiralcel OB-H, hexane:isopropanol = 98:2, flow rate 0.5 mL/min, λ = 254 nm): t_R_ = 12.6 min (minor), t_R_ = 15.9 min (major).

*3-Methoxy-N-(octan-3-yl)aniline* (**6g**). The product (98 mg, 83% yield, 64% ee) was obtained according the general procedure from 3-octanone (71 mg, 0.55 mmol) and *m*-anisidine (62 mg, 0.5 mmol) in 20 h; ^1^H-NMR: δ 0.87 (t, *J* = 7.0 Hz, 3H), 0.91 (t, *J* = 7.5 Hz, 3H), 1.25–1.61 (m, 10H), 3.22–3.28 (m, 1H), 3.44 (brs, 1H), 3.77 (s, 3H), 6.12 (t, *J* = 2.2 Hz, 1H), 6.17–6.22 (m, 2H), 7.05 (t, *J* = 8.1 Hz, 1H); ^13^C-NMR: δ 10.5, 14.5, 23.1, 26.1, 27.7, 32.4, 34.8, 54.5, 55.5, 99.1, 101.8, 106.5, 130.4, 150.0, 161.3; HRMS for C_15_H_26_NO [M+H]^+^: *m/z* Calcd: 236.2009; Found: 236.2008; HPLC (Chiralcel OJ, hexane:isopropanol = 99.5:0.5, flow rate 0.5 mL/min, λ = 254 nm): t_R_ = 21.2 min (major), t_R_ = 25.2 min (minor).

*1-(2,6-Dimethylhept-5-enyl)-3-methoxybenzene* (**6h**) [40]. The product (78 mg, 67% yield, 82% ee) was obtained according the general procedure from 6-methylhept-5-en-2-one (69 mg, 0.55 mmol) and *m*-anisidine (62 mg, 0.5 mmol) in 20 h; ^1^H-NMR: δ 1.17 (d, *J* = 6.3 Hz, 3H), 1.43–1.50 (m, 1H), 1.55–1.64 (m, 1H), 1.59 (s, 3H), 1.69 (d, *J* = 0.8 Hz, 3H), 2.07 (q, *J* = 7.4 Hz, 2H), 3.44 (sextet, *J* = 6.3 Hz, 1H), 3.51 (brs, 1H), 3.76 (s, 3H), 5.10–5.14 (m, 1H), 6.13 (t, *J* = 2.2 Hz, 1H), 6.19 (dd, *J* = 8.0, 2.2 Hz, 1H), 6.23 (dd, *J* = 8.0, 2.2 Hz, 1H), 7.05 (t, *J* = 8.0 Hz, 1H); ^13^C-NMR: δ 18.1, 21.2, 25.1, 26.1, 37.5, 48.6, 55.5, 99.4, 102.3, 106.8, 124.4, 130.4, 132.5, 149.4, 161.3; HRMS for C_15_H_24_NO [M+H]^+^: *m/z* Calcd: 234.1858; Found: 234.1857; HPLC (Chiralcel OD-H, hexane:isopropanol = 98:2, flow rate 0.5 mL/min, λ = 254 nm): t_R_ = 20.5 min (minor), t_R_ = 21.6 min (major).

*N-(1-Cyclopropylethyl)-3-methoxyaniline* (**6i**) [40]. The product (59 mg, 62% yield, 82% ee) was obtained according the general procedure from 1-cyclopropylethanone (46 mg, 0.55 mmol) and *m*-anisidine (62 mg, 0.5 mmol) in 20 h; ^1^H-NMR: δ 0.23–0.34 (m, 2H), 0.43–0.53 (m, 2H), 0.89–0.96 (m, 1H), 1.22 (d, *J* =6.3 Hz, 3H), 2.92-2.99 (m, 1H), 3.76 (s, 3H), 6.15 (t, *J* = 2.2 Hz, 1H), 6.20–6.25 (m, 2H), 7.05 (t, *J* = 8.1 Hz, 1H); ^13^C-NMR: δ 3.0, 3.5, 18.2, 20.6, 53.0, 55.5, 99.6, 102.5, 106.9, 130.3, 149.5, 161.2; HRMS for C_12_H_18_NO [M+H]^+^: *m/z* Calcd: 192.1383; Found: 192.1386; HPLC (Chiralcel OD-H, hexane:isopropanol = 99.5:0.5, flow rate 1 mL/min, λ = 254 nm): t_R_ = 37.0 min (major), t_R_ = 38.0 min (minor). 

*N-(4-(Benzo[d][1,3]dioxol-5-yl)butan-2-yl)-3-methoxyaniline* (**6j**). The product (120 mg, 80% yield, 91% ee) was obtained according the general procedure from 4-benzo[1,3]dioxol-5-yl-butan-2-one (106 mg, 0.55 mmol) and *m*-anisidine (62 mg, 0.5 mmol) in 20 h; ^1^H-NMR: δ 1.19 (d, *J* = 6.3 Hz, 3H), 1.66–1.75 (m, 1H), 1.77–1.86 (m, 1H), 2.63 (t, *J* = 7.8 Hz, 2H), 3.45 (sextet, *J* = 6.3 Hz, 1H), 5.91 (s, 2H), 6.09 (t, *J* = 2.2 Hz, 1H), 6.14–6.17 (m, 1H), 6.22–6.25 (m, 1H), 6.62 (dd, *J* = 7.8, 1.6 Hz, 1H), 6.67 (d, *J* = 1.6 Hz, 1H), 6.72 (d, *J* = 7.8 Hz, 1H), 7.05 (t, *J* = 8.1 Hz, 1H); ^13^C-NMR: δ 21.3, 32.6, 39.5, 48.2, 55.5, 99.4, 101.2, 102.4, 106.7, 108.6, 109.3, 121.5, 130.4, 136.2, 146.0, 148.0, 149.3, 161.3; HRMS for C_18_H_22_NO_3_ [M+H]^+^: *m/z* Calcd: 300.1588; Found: 300.1600; HPLC (Chiralcel OD-H, hexane:isopropanol = 90:10 flow rate 1 mL/min, λ = 254 nm): t_R_ = 25.5 min (major), t_R_ = 29.2 min (minor). 

*N-(4-Methylpentan-2-yl)aniline* (**7a**) [24,46]. The product (71 mg, 80% yield, 88% ee) was obtained according to the general procedure from 4-methylpentan-2-one (55 mg, 0.55 mmol) and aniline (47 mg, 0.5 mmol) in 20 h; ^1^H-NMR: δ 0.91 (d, *J* = 6.6 Hz, 3H), 0.94 (d, *J* = 6.6 Hz, 3H), 1.16 (d, *J* = 6.3 Hz, 3H), 1.26 (dt, *J* = 13.7 Hz, 6.9 Hz, 1H), 1.47 (dt, *J* = 13.7 Hz, 6.9 Hz, 1H), 1.70–1.80 (m, 1H), 3.39 (brs, 1H), 3.47–3.57 (m, 1H), 6.56-6.59 (m, 2H), 6.65 (tt, *J* = 7.3, 1.0 Hz, 1H), 7.13–7.20 (m, 2H);^13^C-NMR: δ 21.5, 23.1, 23.4, 25.5, 46.9, 47.4, 113.5, 117.2, 129.8, 148.2; HRMS for C_12_H_20_N [M+H]^+^: *m/z* Calcd: 178.1596; Found: 178.1590; HPLC (Chiralcel OB-H, hexane:isopropanol = 99.5:0.5, flow rate 0.5 mL/min, λ = 254 nm): t_R_ = 7.7 min (minor), t_R_ = 8.6 min (major). 

*N-(Pentan-2-yl)aniline* (**7b**) [47]. The product (75 mg, 92% yield, 94% ee) was obtained according to the general procedure from 2-pentanone (47 mg, 0.55 mmol) and aniline (47 mg, 0.5 mmol) in 20 h; ^1^H- NMR: δ 0.93 (t, *J* = 7.1 Hz, 3H), 1.17 (d, *J* = 6.2 Hz, 3H), 1.35–1.46 (m, 3H), 1.50–1.60 (m, 1H), 3.45 (brs, 1H), 3.47 (sextet, *J* = 6.2 Hz, 1H), 6.55-6.58 (m, 2H), 6.65 (tt, *J* = 7.3, 1.0 Hz, 1H), 7.13–7.20 (m, 2H); ^13^C-NMR: δ 14.5, 19.7, 21.2, 39.9, 48.6, 113.5, 117.1, 129.7, 148.3; HRMS for C_11_H_18_N [M+H]^+^: *m/z* Calcd: 164.1439; Found: 164.1440; HPLC (Chiralcel OJ, hexane:isopropanol = 99.5:0.5, flow rate 0.5 mL/min, λ = 254 nm): t_R_ = 28.8 min (major), t_R_ = 34.8 min (minor). The ee was determined by weighing the HPLC peaks, as the peak corresponding to the minor enantiomer was too small and wide to be integrated.

*N-(Hexan-2-yl)aniline* (**7c**) [48]. The product (75 mg, 85% yield, 95% ee) was obtained according to the general procedure from 2-hexanone (55 mg, 0.55 mmol) and aniline (47 mg, 0.5 mmol) in 20 h; ^1^H- NMR: δ 0.90 (t, *J* = 7.1 Hz, 3H), 1.16 (d, *J* = 6.3 Hz, 3H), 1.28–1.47 (m, 5H), 1.53–1.56 (m, 1H), 3.41–3.48 (m, 2H), 6.55–6.58 (m, 2H), 6.65 (tt, *J* = 7.3, 1.0 Hz, 1H), 7.13–7.18 (m, 2H); ^13^C-NMR: δ 14.5, 21.2, 23.2, 28.8, 37.4, 48.8, 113.5, 117.1, 129.7, 148.1; HRMS for C_12_H_20_N [M+H]^+^: *m/z* Calcd: 178.1596; Found: 178.1590; HPLC (Chiralcel OB-H, hexane:isopropanol = 99.5:0.5, flow rate 0.25 mL/min, λ = 254 nm): t_R_ = 18.2 min (minor), t_R_ = 19.1 min (major).

*N-(Heptan-2-yl)aniline* (**7d**) [42,49]. The product (79 mg, 83% yield, 92% ee) was obtained according to the general procedure from 2-heptanone (63 mg, 0.55 mmol) and aniline (47 mg, 0.5 mmol) in 20 h; ^1^H-NMR: δ 0.88 (t, *J* = 7.0 Hz, 3H), 1.17 (d, *J* = 6.3 Hz, 3H), 1.26–1.46 (m, 7H), 1.53–1.59 (m, 1H), 3.41 (brs, 1H), 3.44 (sextet, *J* = 6.3 Hz, 1H), 6.55–6.58 (m, 2H), 6.65 (tt, *J* = 7.3 Hz, 1.0 Hz, 1H), 7.13–7.18 (m, 2H); ^13^C-NMR: δ 14.4, 21.2, 23.1, 26.2, 32.3, 37.6, 48.9, 113.5, 117.1, 129.7, 148.3; HRMS for C_13_H_22_N [M+H]^+^: *m/z* Calcd: 192.1752; Found: 192.1754; HPLC (Chiralcel OB-H, hexane:isopropanol = 99.5:0.5, flow rate 0.5 mL/min, λ = 254 nm): t_R_ = 8.8 min (minor), t_R_ = 9.7 min (major). 

*N-(6-Methylhept-5-en-2-yl)aniline* (**7e**). The product (93 mg, 91% yield, 91% ee) was obtained according to the general procedure from 6-methylhept-5-en-2-one (69 mg, 0.55 mmol) and aniline (47 mg, 0.5 mmol) in 20 h; ^1^H-NMR: δ 1.17 (d, *J* = 6.3 Hz, 3H), 1.42–1.51 (m, 1H), 1.59 (s, 3H), 1.55–1.64 (m, 1H), 1.69 (d, *J* = 1.0 Hz, 3H), 2.08 (q, *J* = 7.2 Hz, 2H), 3.43 (brs, 1H), 3.46 (sextet, *J* = 6.3 Hz, 1H), 5.10–5.14 (m, 1H), 6.55-6.58 (m, 2H), 6.65 (tt, *J* = 7.4, 1.0 Hz, 1H), 7.13–7.17 (m, 2H); ^13^C- NMR: δ 18.1, 21.2, 25.1, 26.1, 37.6, 48.5, 113.5, 117.2, 124.4, 129.7, 132.4, 148.2; HRMS for C_14_H_22_N [M+H]^+^: *m/z* Calcd: 204.1752; Found: 204.1749; HPLC (Chiralcel OJ, hexane:isopropanol = 99:1, flow rate 0.5 mL/min, λ = 254 nm): t_R_ = 15.3 min (major), t_R_ = 19.4 min (minor). 

*N-(1-Cyclopropylethyl)aniline* (**7f**). The product (73 mg, 91% yield, 92% ee) was obtained according to the general procedure from 1-cyclopropylethanone (46 mg, 0.55 mmol) and aniline (47 mg, 0.5 mmol) in 20 h; ^1^H-NMR: δ 0.22–0.33 (m, 2H), 0.44–0.53 (m, 2H) 0.88–0.97 (m, 1H), 1.22 (d, *J* = 6.5 Hz, 3H), 2.97 (quintet, *J* = 6.5 Hz, 1H), 3.66 (brs, 1H), 6.57–6.60 (m, 2H), 6.66 (tt, *J =* 7.3 Hz, 1.0 Hz, 1H), 7.13–7.18 (m, 2H);^13^C-NMR: δ 3.0, 3.5, 18.3, 20.6, 52.9, 113.6, 117.3, 129.6, 148.3; HRMS for C_11_H_16_N [M+H]^+^: *m/z* Calcd: 162.1283; Found: 162.1286; HPLC (Chiralcel OJ, hexane:isopropanol = 99.5:0.5, flow rate 0.5 mL/min, λ = 254 nm): t_R_ = 32.4 min (major), t_R_ = 37.3 min (minor). The ee was determined by weighing the HPLC peaks, as the peak corresponding to the minor enantiomer was too small and wide to be integrated.

*N-[4-(2H-1,3-Benzodioxol-5-yl)butan-2-yl]aniline* (**7g**). The product (128 mg, 95% yield, 91% ee) was obtained according to the general procedure from 4-(benzo[1,3]dioxol-5-yl-butan-2-one (106 mg, 0.55 mmol) and aniline (47 mg, 0.5 mmol) in 20 h; ^1^H-NMR: δ 1.20 (d, *J* = 6.3 Hz, 3H), 1.67–1.76 (m, 1H), 1.78–1.87 (m, 1H), 2.64 (t, *J* = 7.8 Hz, 2H), 3.42 (brs, 1H), 3.47 (sextet, *J* = 6.3 Hz, 1H), 5.91 (s, 2H), 6.52–6.55 (m, 2H), 6.62 (dd, *J* = 7.9, 1.7 Hz, 1H), 6.64-6.68 (m, 2H), 6.72 (d, *J* = 7.9 Hz, 1H), 7.12–7.17 (m, 2H); ^13^C-NMR: δ 21.3, 32.6, 39.5, 48.2, 101.2, 108.6, 109.3, 113.6, 117.4, 121.5, 129.7, 136.2, 146.0, 147.9, 148.0; HRMS for C_17_H_20_NO_2_ [M+H]^+^: *m/z* Calcd: 270.1494; Found: 270.1503; HPLC (Chiralcel OD-H, hexane:isopropanol = 98:2, flow rate 1 mL/min, λ = 254 nm): t_R_ = 22.4 min (major), t_R_ = 24.8 min (minor).

*N-(4-(Benzo[d][1,3]dioxol-5-yl)butan-2-yl)-4-bromoaniline* (**7h**). The product (122 mg, 70% yield, 84% ee) was obtained according the general procedure from 4-(benzo[1,3]dioxol-5-yl-butan-2-one (106 mg, 0.55 mmol) and 4-bromoaniline (86 mg, 0.5 mmol) in 30 h; ^1^H-NMR: δ 1.18 (d, *J* = 6.1 Hz, 3H), 1.66–1.84 (m, 2H), 2.62 (t, *J* = 7.5 Hz, 2H), 3.36–3.43 (m, 1H), 3.43 (brs, 1H), 5.92 (s, 2H), 6.39 (d, *J* = 8.6 Hz, 2H), 6.60 (d, *J* = 7.8 Hz, 1H), 6.65 (s, 1H), 6.72 (d, *J* = 7.8 Hz, 1H), 7.21 (d, *J* = 8.6 Hz, 2H); ^13^C-NMR: δ 21.1, 32.5, 39.3, 48.2, 101.2, 108.6, 108.7, 109.3, 115.1, 121.5, 132.4, 136.0, 146.1, 146.9, 148.0; HRMS for C_17_H_19_^79^BrNO_2_ [M+H]^+^: *m/z* Calcd: 348.0599; Found: 348.0592; HPLC (Chiralcel OJ, hexane:isopropanol = 90:10, flow rate 1 mL/min, λ = 254 nm): t_R_ = 46.8 min (major), t_R_ = 65.6 min (minor).

## 4. Conclusions

Together with our previous results [28,30], this paper demonstrates that Cp*Ir(diamine)-phosphate is a powerful catalytic system to enable highly efficient DARA of both aromatic and aliphatic ketones. The catalysis is brought about by the cooperative action of the metal and the counteranion, with the former activating H_2_ whilst the latter ion-pairing with the protonated substrate. Without the counteranion or with mismatched chirality [28] between the anion and the diamine ligand, much reduced enantioselectivities resulted. In this regard, our bifunctional catalysis differs from the original Noyori proposition, in which the diamine ligand provides the second functionality, i.e. the acidic NH_2_ proton. Whether or not the amino group functions in the current DARA remains to be delineated, however, and this is being investigated in our lab.
